# Influenza activity in Cambodia during 2006-2008

**DOI:** 10.1186/1471-2334-9-168

**Published:** 2009-10-15

**Authors:** Sek Mardy, Sovann Ly, Seng Heng, Sirenda Vong, Chea Huch, Chea Nora, Nima Asgari, Megge Miller, Isabelle Bergeri, Sybille Rehmet, Duong Veasna, Weigong Zhou, Takeshi Kasai, Sok Touch, Philippe Buchy

**Affiliations:** 1 Institut Pasteur in Cambodia, 5 Monivong Blvd, Phnom Penh, Cambodia; 2 Communicable Disease Control Department, Ministry of Health, #151-153 Kampuchea Krom Blvd, Phnom Penh, Cambodia; 3 World Health Organization, #177-179 Pasteur Street, Khan Donpenh, Phnom Penh, Cambodia; 4 World Health Organization, Regional Office for the Western Pacific, United Nations, Avenue, PO Box 2932, 1000 Manila, Philippines

## Abstract

**Background:**

There is little information about influenza disease among the Cambodian population. To better understand the dynamics of influenza in Cambodia, the Cambodian National Influenza Center (NIC) was established in August 2006. To continuously monitor influenza activity, a hospital based sentinel surveillance system for ILI (influenza like illness) with a weekly reporting and sampling scheme was established in five sites in 2006. In addition, hospital based surveillance of acute lower respiratory infection (ALRI) cases was established in 2 sites.

**Methods:**

The sentinel sites collect weekly epidemiological data on ILI patients fulfilling the case definition, and take naso-pharyngeal specimens from a defined number of cases per week. The samples are tested in the Virology Unit at the Institut Pasteur in Phnom Penh. From each sample viral RNA was extracted and amplified by a multiplex RT-PCR detecting simultaneously influenza A and influenza B virus. Influenza A viruses were then subtyped and analyzed by hemagglutination inhibition assay. Samples collected by the ALRI system were tested with the same approach.

**Results:**

From 2006 to 2008, influenza circulation was observed mainly from June to December, with a clear seasonal peak in October shown in the data from 2008.

**Conclusion:**

Influenza activity in Cambodia occurred during the rainy season, from June to December, and ended before the cool season (extending usually from December to February). Although Cambodia is a tropical country geographically located in the northern hemisphere, influenza activity has a southern hemisphere transmission pattern. Together with the antigenic analysis of the circulating strains, it is now possible to give better influenza vaccination recommendation for Cambodia.

## Background

Influenza epidemics occur worldwide annually, resulting in considerable morbidity and causing 250,000 to 500,000 deaths annually worldwide [1]. The epidemiology and prevalence of influenza displays a seasonal pattern in temperate regions. Information concerning seasonality and prevalence is crucial for development of effective regional and global seasonal influenza prevention strategies as well as pandemic influenza control measures [2]. Data on the epidemiology of influenza however, are particularly limited in tropical countries [2,3]. To better describe the epidemiology, seasonality and disease burden of influenza as well as to minimize the impact of this disease, the World Health Organization (WHO) formed an Influenza Surveillance Network to detect the emergence and spread of new influenza antigenic variants, to use circulating strains characteristics information to update the formulation of influenza vaccine, and to provide as much warning as possible of the next pandemic. In 1952, WHO established an international laboratory-based surveillance network for influenza. The network currently consists of 125 National Influenza Centre (NIC) laboratories in 95 countries, and four WHO Collaborating Centers for Reference and Research on Influenza (WHO CC) [4].

Prior to the emergence of H5N1 related pandemic threat, influenza data were scarce in Cambodia as laboratory capacities were also limited. To better understand influenza in Cambodia, the NIC was established within a joint collaboration between the Center for Diseases Control Department/Ministry of Health and the Institut Pasteur in Cambodia (IPC) in August 2006. We summarize in this report data gathered from the Cambodian NIC which is based on sampling ILI patients from 5 sentinel outpatients' departments located across the country. Some of these sites were also selected for their proximity to neighboring countries since movement across the borders may result in importation of influenza strains. We also report influenza data during 2007 - 2009 from two hospitals in which IPC has collaborated with clinicians to determine bacterial and viral etiologies of inpatients with acute lower respiratory infections (ALRI).

## Methods

### Geographic and climatic background

Cambodia is a country in Southeast Asia, bordered by Thailand, Laos, Vietnam and the Gulf of Thailand. Cambodia covers 181,035 square kilometers and consists of 27 provinces. As a tropical country, the climate has marked dry and rainy seasons of relatively equal length with averaging temperatures ranging from 24 to 38 degrees Celsius. The dry season runs generally from November to April and the rainy season starts in May-June and ends in October-November.

### Study sites

The ILI surveillance encompasses 5 outpatients' departments located in the following provincial or referral hospitals: Takeo (southern Cambodia), Battambang (western Cambodia), Kampong Cham (central-north Cambodia), a pediatric hospital in Siem Reap (north-west Cambodia) and the National Pediatric hospital in Phnom Penh capital (central Cambodia). Since April 2007, the Institut Pasteur in Cambodia (IPC) has also collaborated with Takeo and Kampong Cham provincial hospitals to study the etiologies of acute lower respiratory infections among hospitalized patients. Each sentinel and study site was required to randomly collect nasopharyngeal swabs of 5 to 10 ILI patients per week all year long. For the hospital study we obtained nasopharyngeal samples from all hospitalized patients admitted to the two hospital sites collaborating with ALRI study. Swab specimens were immediately introduced into a sterile tube containing virus transport medium (VTM), kept in liquid nitrogen containers and transported to the Institut Pasteur in Phnom Penh on a weekly basis.

### Surveillance and case definition

Multiple respiratory viruses, including respiratory syncytial virus, parainfluenza viruses, adenovirus, and rhinovirus, can mimic influenza symptoms [5]. Since the middle of 2008, the following WHO definition of ILI was used: person with sudden onset of fever >38°C armpit and cough or sore throat in the absence of other diagnosis [6]. From 2006 to early 2008, the ILI case definition was identical except for the fever criteria which was set at ≥ 37.5°C armpit temperature. For the ALRI study, all patients were recruited between April 2007 and March 2009. A case for the under 5 age group was defined as an illness of <10 days duration that consisted of cough or difficult breathing plus tachypnea. For the 5-14 age group, case definition included the above signs and symptoms plus fever > 38°C armpit on admission. For the 15 and over age group, a case was defined as anyone with fever > 38°C armpit on admission plus tachypnea or chest pain or auscultatory crackles. Clinical examination of cases was conducted by hospital clinicians and recorded alongside demographic information by means of a standard case report form and chest-X rays were systematically requested.

### Laboratory Methods

The collected naso-pharyngeal samples were immediately frozen at the hospital in liquid nitrogen and the container was sent on a weekly basis to the IPC's Virology Unit where specimens were stored at -80°C prior to testing. Viral RNA was extracted from 140 μL of VTM by using the QIAamp Viral RNA Isolation Kit (QIAGEN, CA, USA) and amplified by multiplex reverse transcriptase-polymerase chain reaction (RT-PCR) detecting simultaneously influenza A virus, influenza B virus, respiratory syncytial virus and human metapneumovirus, as previously reported [7]. Influenza A viruses were subsequently subtyped by conventional RT-PCR methods targeting specifically H1, H3, N1 and N2 genes (protocols available upon request to authors). The influenza positive specimens were also inoculated onto MDCK cells as previously described [8]. The influenza strains were analyzed by a hemagglutination inhibition (HAI) test using a WHO influenza diagnostic kit [9]. A representative number of influenza isolates was sent each year to the WHO Collaborating Center in Melbourne, Australia, for further analysis (Table 1).

**Table 1:** Breakdown of influenza positive isolates by type and subtype in 2006 - 2008

Year	No. of specimens	No. of positives (%)	Type		Subtype of influenza A			WHOCC, Melbourne*		
					
			Influenza A (%)	Influenza B (%)	H1N1 (%)	H3N2 (%)	H5N1 (%)	H1N1	H3N2	Influenza B
2006	516	30 (5.8)	30 (100)	0	0	30 (100)	0	N/A	26	N/A
2007	1827	104 (5.7)	44 (42.3)	60 (57.7)	14 (31.8)	29 (65.9)	1 (2.3)	19	24	33
2008	2673	259 (9.7)	171 (66)	88 (34)	31 (18.1)	139 (81.3)	1 (0.6)	3	18	6

* Number of specimens sent to the WHO Collaborating Center in Melbourne from 2006 to 2008.

### Statistical analyses

The comparisons between percentages and two means were tested by Chi2 test and Student's t test respectively. A p value < 0.05 was considered significant. Proportions, means and all statistical analyses were performed using STATA 9.0 (Statacorp., college station, TX, Texas).

## Results

**Figure 1:**
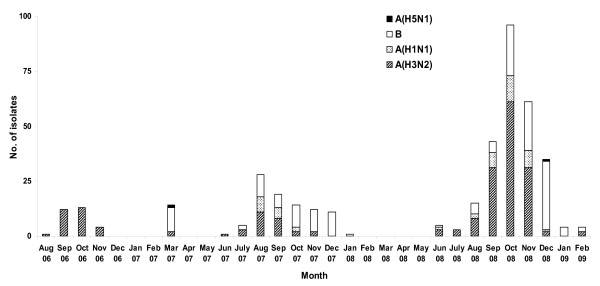
**Monthly distribution of influenza isolates from late 2006 to early 2009**.

The number of influenza strains identified during the 2006-2008 period by both ILI and ALRI systems are detailed in Table 1 and Figure 1.

ILI surveillance program tested a total of 3,148 samples during 2006-2008. The average age of the studied population was 11.9 years (range, 1 month-76 years) and 50.8% were male. Of these, 338 (10.7%) tested positive. The average age of positive patients was 8.7 years (range, 1 month to 76 years) and 180 (53.3%) were male. There were no differences in age and gender distribution across the three testing years. However, the influenza-infected patients were significantly younger than those tested negative for influenza viruses (8.7 versus 11.9 years; p < 0.001). Overall positive rates varied from 5.8% in 2006, 7.7% in 2007 and 15.3% in 2008.

The average age among the patients included in the ALRI study was 38.2 years (range: 1 month to 87 years) and 61.8% were male. Of the 1,868 patients admitted with ALRI during 2007-2008, only 64 (3.4%) tested positive for influenza virus - 1.4% in 2007 and 3.6% in 2008. The average age of positive cases was 15.2 years (range, 3 months to 82 years); 28 (51.7%) were male. The patients with influenza infection were significantly younger than the other patients recruited in the ALRI study (p < 0.001).

The difference between both ILI and ALRI system for influenza detection was significant (p < 0.001).

From 2006 to 2008, influenza infections were observed mainly from June to December (Figure 1). However, some influenza activity (mainly influenza B, but not exclusively) was also detected in March 2007. During this time a human case of influenza A/H5N1 infection was also identified by the NIC in a patient admitted in a reference hospital of the capital for severe pneumonia. Incidently, the H5N1 case detected in 2008 was identified during a fever surveillance study (Wierzba T, personal communication).

**Figure 2:**
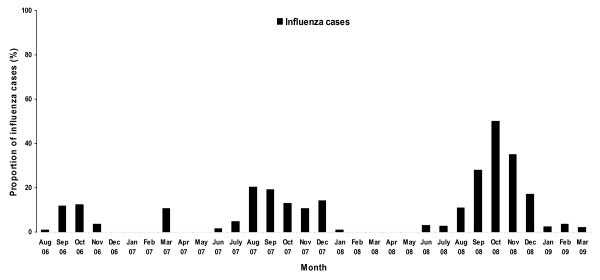
**Monthly proportion of positive influenza samples among ILI specimens tested from late 2006 to early 2009**.

**Figure 3:**
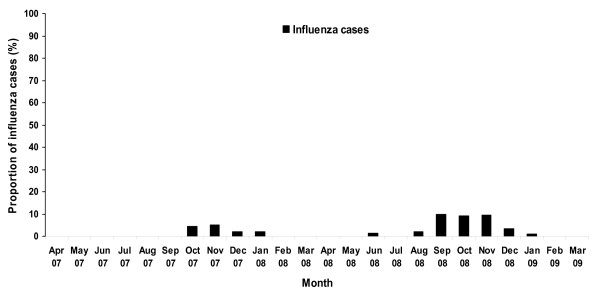
**Monthly proportion of positive influenza samples among ALRI specimens tested from 2007 to early 2009**.

The proportion of influenza virus detected among ILI specimens from 2006 to early 2009 varies between 0% in April and May and 50% in October 2008 (Figure 2). Influenza positivity rates varied between the sentinel sites: 40% in the National Pediatric hospital in Phnom Penh, 9.5% in Takeo, 20% in Kampong Cham, 16% in Battambang and 14.5% in a pediatric hospital in Siem Reap. Only 5 to 10% of the ALRI specimens were positive for influenza virus during the highest transmission season (from September to November) (Figure 3).

The antigenic analysis show that influenza A/H1N1 isolates belonged to the A/New Caledonia/20/99-like group in 2007 and A/Brisbane/59/2007-like group in 2008. In 2006 and 2007, A/H3N2 strains tested were close to A/Wisconsin/67/2005-like, but in 2008 the strains drifted to A/Brisbane/10/2007-like. The influenza B strains were B/Malaysia/2506/2004-like in 2007 and B/Florida/4/2006-like in 2008. These data suggest that the influenza strains circulating in Cambodia from 2007 to 2008 match with the strains contained in the influenza vaccine recommended for the southern hemisphere (Table 2). Nevertheless, in 2006, only H3N2 viruses were isolated in Cambodia and these strains belonged to the A/Wisconsin/67/2005-like group while the seasonal vaccine for the southern hemisphere contained the A/California/7/2004-like strain (Table 2).

**Table 2:** Comparison between vaccine strains and circulating influenza strains in Cambodia from 2006 to 2008.

Season	A/H1N1		A/H3N2		Influenza B	
	**Vaccine stain**	**Cambodian strains**	**Vaccine strain**	**Cambodian strains**	**Vaccine strain**	**Cambodian strains**

**2006**	A/New Caledonia/20/1999-like	N/A	A/California/7/2004-like	A/Wisconsin/67/2005-like	B/Malaysia/2506/2004-like	N/A

2006-2007	A/New Caledonia/20/1999-like		A/Wisconsin/67/2005-like		B/Malaysia/2506/2004-like	

**2007**	A/New Caledonia/20/1999-like	A/New Caledonia/20/1999-like	A/Wisconsin/67/2005-like	A/Wisconsin/67/2005-like	B/Malaysia/2506/2004-like	B/Malaysia/2506/2004-like

2007-2008	A/Solomon Islands/3/2006-like		A/Wisconsin/67/2005-like		B/Malaysia/2506/2004-like	

**2008**	A/Solomon Islands/3/2006-like	A/Brisbane/59/2007-like	A/Brisbane/10/2007-like	A/Brisbane/10/2007-like	B/Florida/4/2006-like	B/Florida/4/2006-like

2008-2009	A/Brisbane/59/2007-like		A/Brisbane/10/2007-like		B/Florida/4/2006-like	

Bolded seasons correspond to the southern hemisphere.

## Discussion

To our knowledge, this is the first report of influenza activity in Cambodia. It appears that there is a consistent pattern of influenza seasonality during these 3 years, with detection peaking during the rainy season from June to December. During the hot and dry seasons there was little to no detection of influenza, apart from an unusual peak during March 2007, a period where increased influenza activity was detected at all 5 sentinel sites. This overall pattern contrasts with that of Northern Vietnam, Thailand or Singapore in which influenza viruses circulate year-round and appears more similar to that of countries of southern hemisphere (Australia, New Zealand) [10-13]. The reasons for the seasonality of influenza are not clearly known and are probably the result of a combination of multiple factors as, for instance, climate conditions, epidemiology (population's age, density, migration, etc.), host susceptibility and virus characteristics [14]. Others also reported that influenza seasonality coincides with rainy season [15-18] and that temperature and humidity play a more important role than population factors in driving seasonality of transmission [18,19]. Importantly, the role of influenza transmission in schools (and subsequently to households) is probably very limited in Cambodia because they are closed from July to September [20].

This study provides data useful for accurate recommendations on influenza vaccination. People living in Cambodia would ideally receive vaccines by April - May in order to develop protective immunity before the peak season of transmission.

We were able to also identify severe influenza infections among hospitalized patients paralleling the same seasonality as observed within the NIC's ILI surveillance. To our surprise, few cases were detected among hospitalized patients with ALRI. We believe that this low frequency of cases may not be a reflection of the extent of influenza-related severity, rather that it is explained by the delay in hospital admission of Cambodian patients presenting with severe respiratory infections. Indeed, most bacterial lung infections included in our hospital study were diagnosed among patients who attended hospital too late after the onset of respiratory symptoms for viral detection (average, 6 days; IPC's unpublished data). It is plausible that many of these bacterial infections were secondary infections from an initial influenza disease. Further testing for influenza using serology should be performed among patients with bacterial respiratory infections during the seasonal epidemics to establish this hypothesis. In addition, the average age of the patients presenting to hospital with ALRI was significantly higher (p < 0.001) than patients with ILI symptoms since influenza is mainly found in children, it may explain the rate differences between both studies. In 2006, few influenza viruses were detected, as the NIC and its sentinel sites were just established in August 2006. In this year, all influenza-positive isolates belonged to influenza A/H3N2 sub-type which is consistent with influenza activity reported worldwide by WHO. Indeed, in 2006, a low influenza activity was observed and influenza A/H3N2 virus was the predominant subtype in many European and Asian countries [21]. In 2007, both influenza A subtypes as well as influenza B viruses co-circulated at comparable level. Among others, outbreaks of influenza B virus were also reported in 2007 in China, Hungary and in the United States [22]. In 2008, influenza A and B viruses were co-circulating as well but influenza A viruses represented two thirds of the strains detected. Similar general circulation patterns were observed worldwide in 2008 and, as in Cambodia, H3N2 subtype was responsible for most outbreaks in some European countries or in Japan [23].

Since the establishment of the NIC and the ILI surveillance system, we observed a regular increase in the influenza positivity rates in the samples that were tested. This could be linked to a higher influenza activity but also to an improvement of the system from patient recruitment (with adjustment of the case definition in 2008 for the fever criteria), to sample collection and transport to testing. Significant differences in the influenza positivity rate were observed between the sentinel sites. It was expected that paediatric hospitals will have a higher positive rate than adult hospitals, but interestingly, comparing the two paediatric hospitals, the one based in Phnom Penh produced more influenza-positive samples than the hospital located in Siem Reap, perhaps emphasizing the important role of the physician in the successful recruitment of patients who are likely to have influenza infection.

There are some limitations to the sentinel surveillance data. Sentinel data cannot be extrapolated precisely to the rest of the population, as the outpatients clinic serving sentinel sites were not truly representative. Random selection of patients is not yet well standardized and depends on the goodwill of physicians in participating in influenza surveillance. In addition, ILI consultation rates were not provided, and data is lacking on the catchment areas of the sentinel sites. As a result, little inferences can be made on disease burden or severity in Cambodia in general. Despite these problems, the system has been useful in meeting the purposes of influenza surveillance, identifying the predominant circulating strains in the community and allowing the formulation of guidelines for influenza vaccine composition for the subsequent year.

Since 2005, a total of eight human cases of influenza A/H5N1 have been identified in Cambodia [24]. Interestingly, H5N1 human cases (e.g. in March 2007 and December 2008) were detected while there was evidence of background human influenza activities. As in other Asian countries, there is some evidence of endemicity of avian influenza H5N1 circulation in poultry in Cambodia [25]. There is little evidence for human-to-human transmission of the H5N1 virus. Additional mutations or re-assortment events are probably required to permit efficient human-to-human spread which could trigger a pandemic [26,27]. Thus, if H5N1 virus infection in humans occurs during these seasonal epidemics, the risk of re-assortment increases.

Even though there is little evidence of mild or asymptomatic H5N1 human infections, the increasing incidence of cases with influenza-like symptoms have been reported more frequently since 2005 [28]. In most countries, H5N1 testing is usually limited to symptomatic cases with a recent history of exposure to dead or sick poultry. Thus, if the H5N1-infected patients deny contact with the usual animal source of contamination, hospitalization and testing would be unlikely and the symptoms could be mistaken for seasonal flu. Others may not seek medical attention, assuming that the H5N1 infection is mild and does not require medical attention, leading to exposure of others. Hence, H5N1 virus detection by NICs should be systematic.

## Conclusion

This study provides the first data regarding influenza activity in Cambodia, combining influenza-like illness surveillance and acute lower respiratory infection surveillance. It demonstrates that even though Cambodia is a tropical country geographically located in the northern hemisphere, influenza activity has a southern hemisphere transmission pattern. Together with the antigenic analysis of the circulating strains, it is now possible to provide better recommendations for influenza vaccination in Cambodia. The ILI surveillance system produced a higher number of seasonal influenza cases than the ALRI surveillance system. This system is therefore more useful for maximizing detection of seasonal influenza cases to generate data regarding vaccine composition, and antiviral susceptibility. Significantly, the parallel circulation of seasonal and avian influenza viruses represents a risk for pandemic by re-assortment during human co-infection. This highlights the importance to reinforce and develop the influenza surveillance systems (both ILI and ALRI); especially in countries where H5N1 virus infection has become endemic in poultry.

## Competing interests

The authors declare that they have no competing interests.

## Authors' contributions

SM performed analysis of the data and drafted the manuscript. SV, SL, SH, CH, CN, NA, MM, IB, SR, WZ, TK and ST participated in the design and coordination of the study. SV and DV revised the manuscript and did complementary analysis. PB conceived the study, participated in its design and coordination, and helped to draft the manuscript. All authors read and approved the final version of the manuscript.

## Pre-publication history

The pre-publication history for this paper can be accessed here:

http://www.biomedcentral.com/1471-2334/9/168/prepub
